# CRISPR-Cas9 mediated RALA knockout and reconstitution: insights into the detection and role of RALA S194 phosphorylation in Ras-dependent and Ras-independent cancers

**DOI:** 10.1242/bio.061884

**Published:** 2025-07-21

**Authors:** Mayuresh Vishwas Konde, Siddhi Inchanalkar, Tushar Manik Sherkhane, Nilesh Deshpande, Mishika Virmani, Kajal Singh, Nagaraj Balasubramanian

**Affiliations:** IISER Pune: Indian Institute of Science Education Research Pune, India

**Keywords:** RALA, CRISPR-Cas9, pS194RALA, V_MLN_, RAS-dependent, RAS-independent

## Abstract

Downstream of oncogenic RAS, RALA is critical for cancer tumorigenesis, possibly regulated by phosphorylation of its Serine194 residue. We made CRISPR-Cas9 RALA knockout (RALA KO) in three RAS-dependent and two RAS-independent cancer cells. Detection of RALA S194 phosphorylation using the commercial anti-phospho-RALA antibody lacks specificity in all three RAS-dependent cancers. siRNA knockdown of RALA and AURKA inhibition by MLN8237 (V_MLN_) also did not affect pS194RALA detection in these cancers. RALA KO MiaPaCa2 (RAS-dependent) and MCF7 (RAS-independent) cells, stably reconstituted with WT-RALA and S194A-RALA mutants, showed no effect on RALA activation. Tumor growth was, however, restored partly by WT-RALA, but not S194A-RALA mutant. Thus, RALA S194 phosphorylation is needed for tumor formation, not affecting its activation, but possibly through its localization.

## Teaser

**Summary:** This study gives insight into the novel role of RALA S194 phosphorylation in regulating tumor formation in cancer cells. It also shows that this regulation of tumor formation comes from its role in RALA localization rather than RALA activation.

## INTRODUCTION

RALA regulates cell migration, proliferation, cell spreading, anchorage independent growth (AIG) and tumorigenesis. Its function depends upon its localization and activation in the cell and is mainly regulated by RALGEFs. In recent years, AURKA has emerged as a novel regulator of RALA (Lim et al., 2010). AURKA is a well-known mitotic kinase involved in centrosome maturation (Nikonova et al., 2013). *In-vitro* kinase assay shows it phosphorylates RALA at Serine 194 residue (S194) but not RALB (Wu et al., 2005). AURKA-mediated phosphorylation of RALA at S194 regulates its localization at cell membranes (Lim et al., 2010). It remains unclear, and possibly context-dependent, whether AURKA-mediated RALA phosphorylation at S194 can regulate RALA activation. PKA could also mediate RALA phosphorylation at S194 (Neel et al., 2014).

Studies in non-transformed HEK293 and MDCK cells expressing constitutively active RALA^V23^ and phosphodeficient S194A-RALA^V23^ mutants show this phosphorylation to be vital for RALA-dependent AIG (Wu et al., 2005; Lim et al., 2010). In some pancreatic cancer cells, the knockdown of RALA and reconstitution with WT-RALA and S194A-RALA mutants further support this claim in AIG studies (Lim et al., 2010). AURKA inhibitor (MLN8237) treatment of pancreatic and breast cancer cells also affect their AIG (Neel et al., 2014; Inchanalkar et al., 2018).

The only known tumor formation study in RALA knockdown pancreatic cancer cells (CFPac-1, HPAC, Capan1) reconstituted with WT-RALA versus S194A-RALA phosphodeficient mutant showed loss of RALA to affect tumor formation that is restored by WT-RALA reconstitution. S194A-RALA mutants, however, had variable effects and were comparable to WT-RALA in CFPac-1, comparable to RALA KD in Capan-1, and better than RALA KD but not equivalent to WT-RALA in HPAC tumors (Lim et al., 2010). Targeting AURKA activation using inhibitors like MLN8237 (Alisertib) in pancreatic cancers (PancT4 cell line and Primary cells from tumors) suppresses tumor formation (Neel et al., 2014). However, this study showed no change in RALA S194 phosphorylation detected by western blot using anti-phospho-RALA (Merck-Millipore, 07-2119), suggesting the effects seen might be independent of RALA. These studies were exclusively done in RAS-dependent cancers.

These differences in AURKA-RALA crosstalk observed in non-transformed versus cancer cells could, in part, be mediated by regulatory influencers, like the presence of oncogenic RAS. Oncogenic RAS-mediated regulation of the RAL GTPases is vital for RAS-mediated transformation and is seen to drive AIG (Lim et al., 2010). RAS-dependent regulation of RAL is known to be mediated by RAS-dependent RALGEFs (Neel et al., 2011). Constitutively active RAS is also seen to promote AURKA expression in cancer cells (dos Santos et al., 2016). RAS is also known to interact with AURKA (Umstead et al., 2017) and joint expression of both is further seen to promote AIG (Tatsuka et al., 2005). This study, hence, evaluates the role RALA S194 phosphorylation has in cancers and what influence the presence of oncogenic RAS could have on its regulation and role. In doing so, it also helps us comment on how the detection of pS194RALA could have implications in interpreting the role of this phosphorylation in cancer cells.

## RESULTS

### CRISPR-Cas9 mediated stable knockout of RALA

**Fig. 1.**
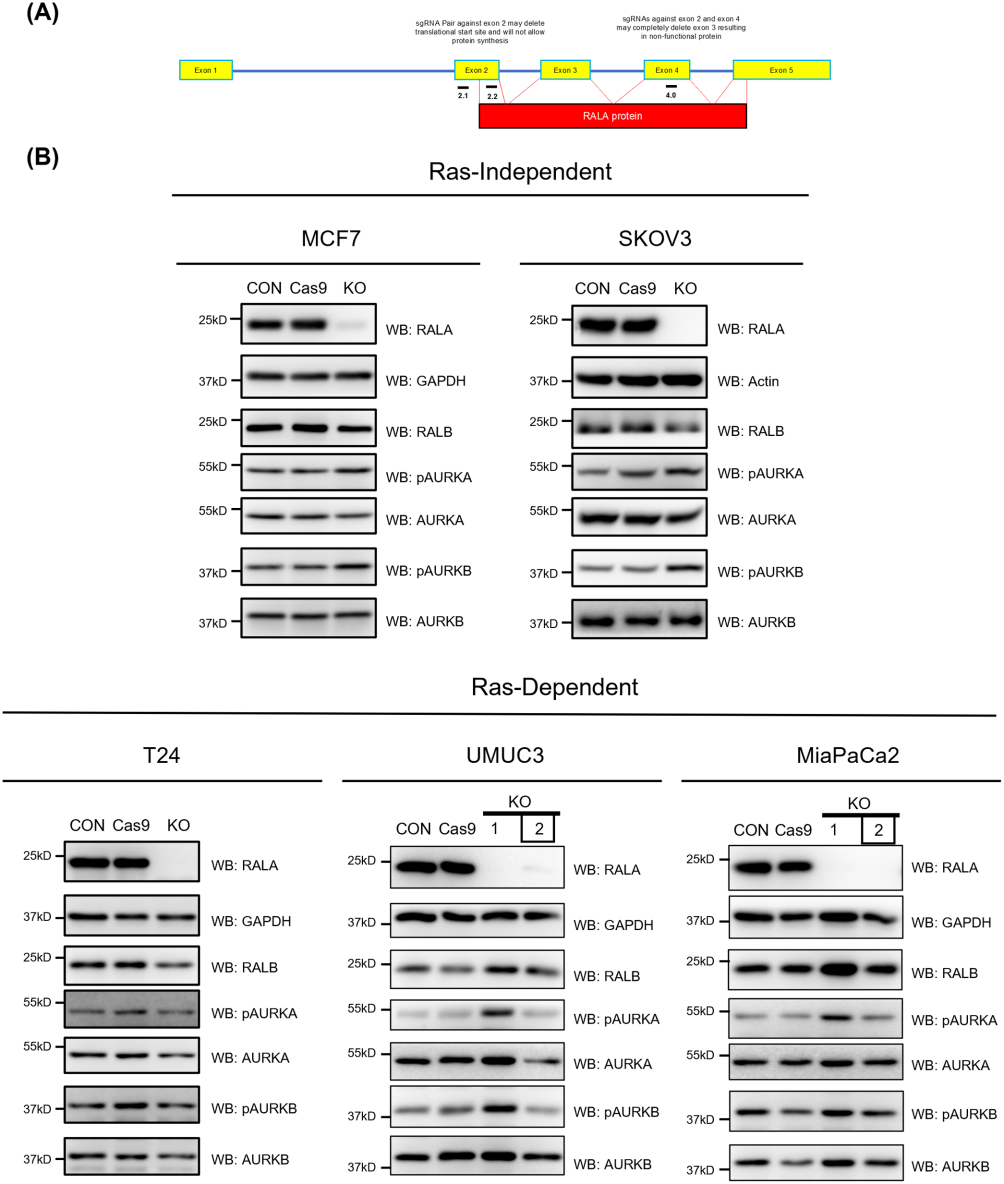
**CRISPR-Cas9 mediated knockout of RALA in RAS-independent and RAS-dependent cancer cells.** (A) The schematic shows an exon map (yellow boxes) for RALA, marking the location of sgRNAs designed against exon 2 (2.1 and 2.2) and exon 4 (4.0) to target RALA. The red box represents the functional RALA protein coded by these exons. (B) Western blot detection (WB) of RALA, RALB, AURKA, AURKB in untreated control (CON), Cas9 control (Cas9), RALA knockout (KO) clones of RAS-independent (MCF7 and SKOV3) (single clone each) and RAS-dependent (T24, UMUC3 and MiaPaCa2) (two clones for some) cells. Clone 2 selected based on these studies is marked by a box. This was accompanied by the western blot detection (WB) of active AURKA (pAURKA – pThr288 AURKA) and active AURKB (pAURKB – pThr232 AURKB). Lysates were further probed for additional proteins of interest, with the results shown in Fig. 2A and Fig. S1B. Blots were reused where required, and the RALA and GAPDH blots from B are also shown for comparison in Fig. 2A.

As a first step to understanding RAS-dependent regulation of RALA S194 phosphorylation and its role in cancers, RAS-independent (MCF7 and SKOV3) and RAS-dependent (T24, UMUC3 and MiaPaCa2) cell lines were chosen for study based on their known RALA expression and activation status (Lim et al., 2010; Pawar et al., 2016; Oxford et al., 2005). We decided to use CRISPR-Cas9 to make stable knockouts of RALA in these cells, allowing us to later reconstitute them with WT-RALA and phosphodeficient (S194A) or phosphomimetic (S194D) mutants. Using the CRISPR-Cas9 approach, we designed two sgRNAs against exon 2 and one against exon 4 of RALA using the Chop-chop tool (Fig. 1A).

**Fig. 2.**
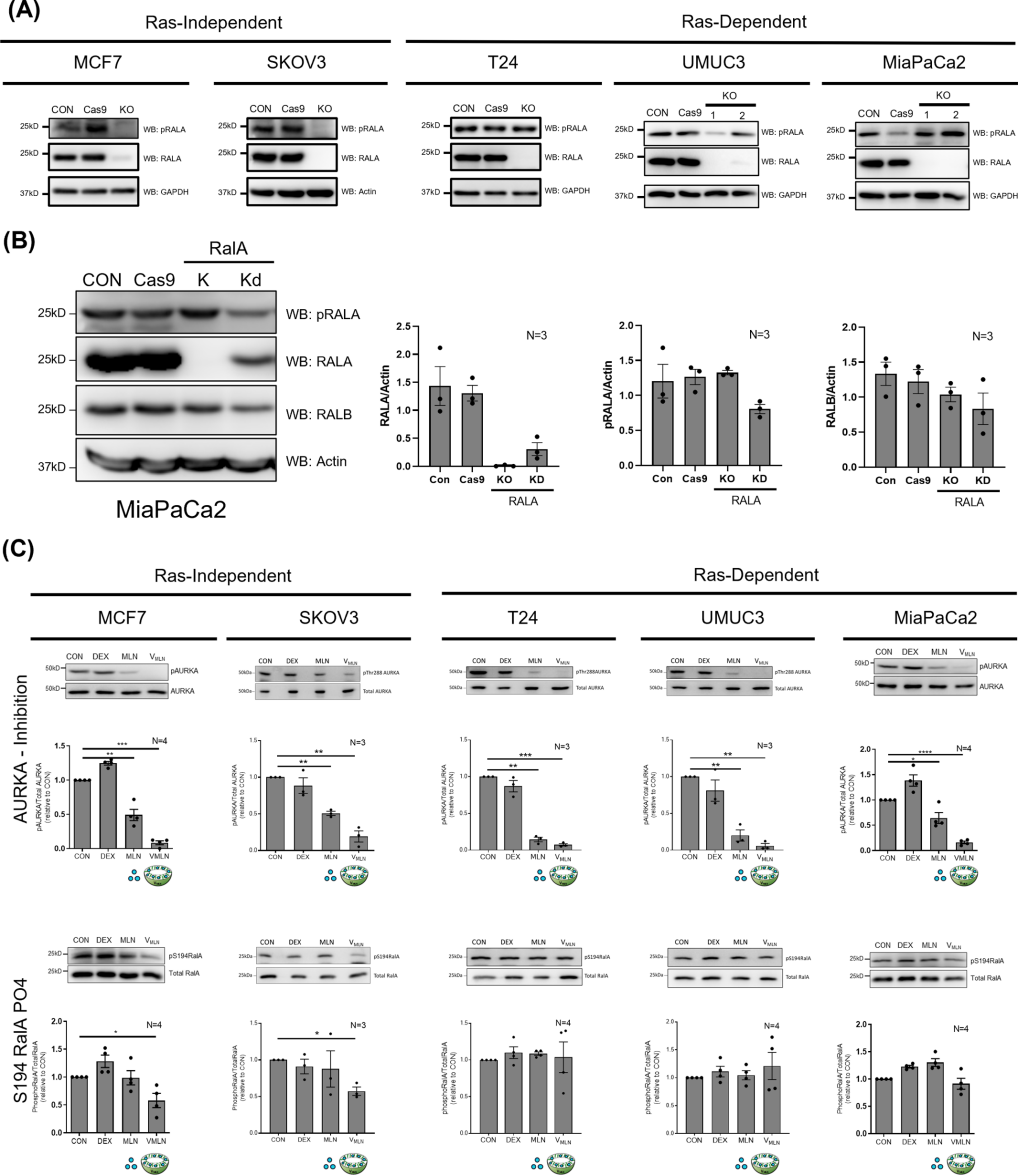
**pS194 RALA detection in RAS-independent and RAS-dependent cancer cells.** (A) Western blot detection of pRALA (RALA S194 phosphorylation) in cell lysate from untreated control (CON), Cas9 control (Cas9) and RALA KO cells using anti-phospho-RALA Antibody (Ser194) (Merck-Millipore, Cat no- 07-2119) in RAS-independent (MCF7 and SKOV3) and RAS-dependent (T24, UMUC3 and MiaPaCa2) cancer cells. pRALA detection in RALA KO clones was part of the experiment presented in Fig. 1B, and hence the RALA and housekeeping protein blots from Fig. 1B are shown here for comparison. (B) Western blot detection of pRALA (RALA S194 phosphorylation) in the cell lysate of control (CON), Cas9 control (Cas9), siRNA mediated RALA KD and CRISPR-Cas9 RALA KO MiaPaCa2 cells. Blots were also probed for RALA, RALB and actin. Blots were quantitated, and the ratio of band intensities for RALA, pRALA and RALB normalized to actin as mean±s.e. from three independent experiments. Statistical analysis was done using the Mann–Whitney test, compared to control *P*-values, if significant, are represented in the graph (*P<0.05, **P<0.01, ***P<0.001). (C) Western blot detection (upper panel) and quantitation (lower panel) of phosphorylation of Threonine 288 residue (pThr288) AURKA, total AURKA, phosphorylation of RALA Serine 194 residue (pRALA) and total RALA, from lysates of MCF7, SKOV3, T24, UMUC3 and MIAPaCa2 treated for 48 h with DMSO (CON) and empty nano-vesicle scaffold (DEX), 0.02μM Free MLN (MLN) and 0.02μM encapsulated MLN (V_MLN_). The ratio of pAURKA/AURKA and pRALA/TotalRALA in each cell line was normalized to their respective controls (CON) (equated to 1) and were represented in the graph as mean±s.e. from at least three independent experiments. Statistical analysis was done using one-sample *t*-test, compared to control *P*-values, if significant, are represented in the graph (*P<0.05, **P<0.01, ***P<0.001).

*In-silico* checks confirmed their lack of off-target binding and efficiency in making double-strand breaks with Cas9. All sgRNAs were then cloned into the pSpBBCas9-Puro vector. These plasmids were sequenced to confirm the presence of the inserted sgRNAs at the right site (Fig. S1A). When used together, these three sgRNAs could support a more complete knockout of RALA, without affecting RALB.

Cancer cells were transfected with all three sgRNAs and the pSpBBCas9-GFP vector (as a marker for transfection), single cells were sorted, clones expanded and tested for RALA expression. Preliminary screening for RALA versus RALB expression allowed us to select clone(s) with the best targeting of RALA without affecting RALB (Fig. 1B). One clone, each for MCF7, SKOV3 and T24 and two clones for UMUC3 and MiaPaCa2, were then probed for the expression of RALA, RALB, and regulatory players that could be affected by this targeting (Fig. 1B). Together, this confirmed that the loss of RALA in MCF7, SKOV3, T24, UMUC3 (clone 2), and MiaPaCa2 (clone 2) without affecting the expression of RALB, AURKA, AURKB (Fig. 1B), AKT, ERK and AMPK (Fig. S1B). No detectable change in the activation of AURKA, AURKB (Fig. 1B), AKT, ERK and AMPK (Fig. S1B) was seen, making them suitable for further evaluation.

PCR using genotyping primers to detect a physical break in exon 2 and/or exon 4 of RALA (reflected as a loss or shift in the size of the PCR amplicon) confirmed the KO status of the selected clones for MCF7, T24, UMUC3 (clone 2) and MiaPaCa2 (clone 2) (Fig. S1C). MCF7, T24, UMUC3 and MiaPaCa2 showed a cut in exon 2, while MCF7 and MiaPaCa2 also had a cut in exon 4. The lack of a detectable loss or shift in amplicon for UMUC3 (clone 1) and MiaPaCa2 (clone 1) ruled them out for further use (Fig. S1C). Despite multiple attempts with multiple primers for genotyping PCR in SKOV3 cells, no amplicon was detected. This inexplicable outcome also ruled out using SKOV3 in further studies (Fig. S1C). This now gives us a confirmed RALA KO clone for MCF7 (RAS-independent) and T24, UMUC3, and MiaPaCa2 (RAS-dependent) cell lines to take forward in these studies.

### Detection of RALA S194 phosphorylation in RAS-independent and RAS-dependent cancer cell lines

Our first objective in using the CRISPR-Cas9 RALA KO cells was to test the detection of RALA phosphorylation at the Serine 194 residue across these cell lines. Use of RALA lacking knockout clones, we hoped will help establish the specificity of the anti-phospho-RALA Antibody (Serine 194 residue) (Merck-Millipore, 07-2119), which has been vital in understanding its role and regulation in earlier studies. The anti-phospho-RALA Antibody (Ser194) from MERCK-Millipore is raised against KLH-conjugated linear peptide corresponding to human RALA phosphorylated at Serine 194. This antibody is used across different studies (Neel et al., 2014) and cancer cell lines have been reported to occasionally detect pS194 RALA in cancer cells even when RALA is knocked down (Neel et al., 2014). This raises questions about its specificity. In previous studies, such an evaluation has not been discussed, making it difficult to know whether such specificity issues exist across cell types. We were in a unique position to evaluate this.

Cell lysates from confirmed RALA KO clones of MCF7, T24, UMUC3 and MiaPaCa2 (evaluated in Fig. 1) were probed with the anti-phospho-RALA Antibody (Ser194) and showed variable results. No detectable band for pS194 RALA was seen in RALA KO Ras-independent MCF7 cells (Fig. 2A). In SKOV3 cells, despite genotyping issues, a near-complete loss of RALA was detected in western blot studies. We tested and saw no detectable band for pS194 RALA in RALA lacking SKOV3 cells (Fig. 2A). However, in RAS-dependent T24, UMUC3 and MiaPaCa2 cells, despite the visible absence of RALA in the KO clones, a distinct band at the expected size was consistently detected by the pS194 RALA antibody (Fig. 2A). This was comparable to the pS194RALA band detected in their respective control cells. To confirm this observation, we also used a RALA-specific siRNA (Pawar et al., 2016) to knockdown RALA in MiaPaCa2 cells and tested the detection of pS194RALA in this lysate. Despite the siRNA causing a ∼80% drop in RALA protein levels, a distinct band at the expected size continued to be detected by the phospho-RALA antibody, comparable in intensity to control (Fig. 2B).

As an additional approach to evaluate this specificity issue, we tested for the effect inhibition of AURKA (known to phosphorylate RALA at S194) has on the band detected by the phospho-RALA antibody. Previous studies from the lab have developed a dextran nano-vesicle encapsulated MLN8237 (V_MLN_), which explicitly inhibits AURKA without affecting AURKB (Inchanalkar et al., 2018). V_MLN_ uptake in MCF7, SKOV3, T24, UMUC3 and MiaPaCa2 cells was confirmed using Rhodamine (RhB) encapsulated nano-vesicles (Fig. S2A). V_MLN_-mediated inhibition of AURKA (pThr288) was better than free MLN8237 across these cell lines (Fig. 2C). Interestingly, AURKA inhibition causes a significant decrease in detectable pS194RALA in MCF7 and SKOV3 cell lysate but not in T24, UMUC3 and MiaPaCa2 cells relative to control cells (Fig. 2C), further questioning the specificity of phospho-RALA antibody in T24, UMUC3 and MiaPaCa2 cells.

**Fig. 3.**
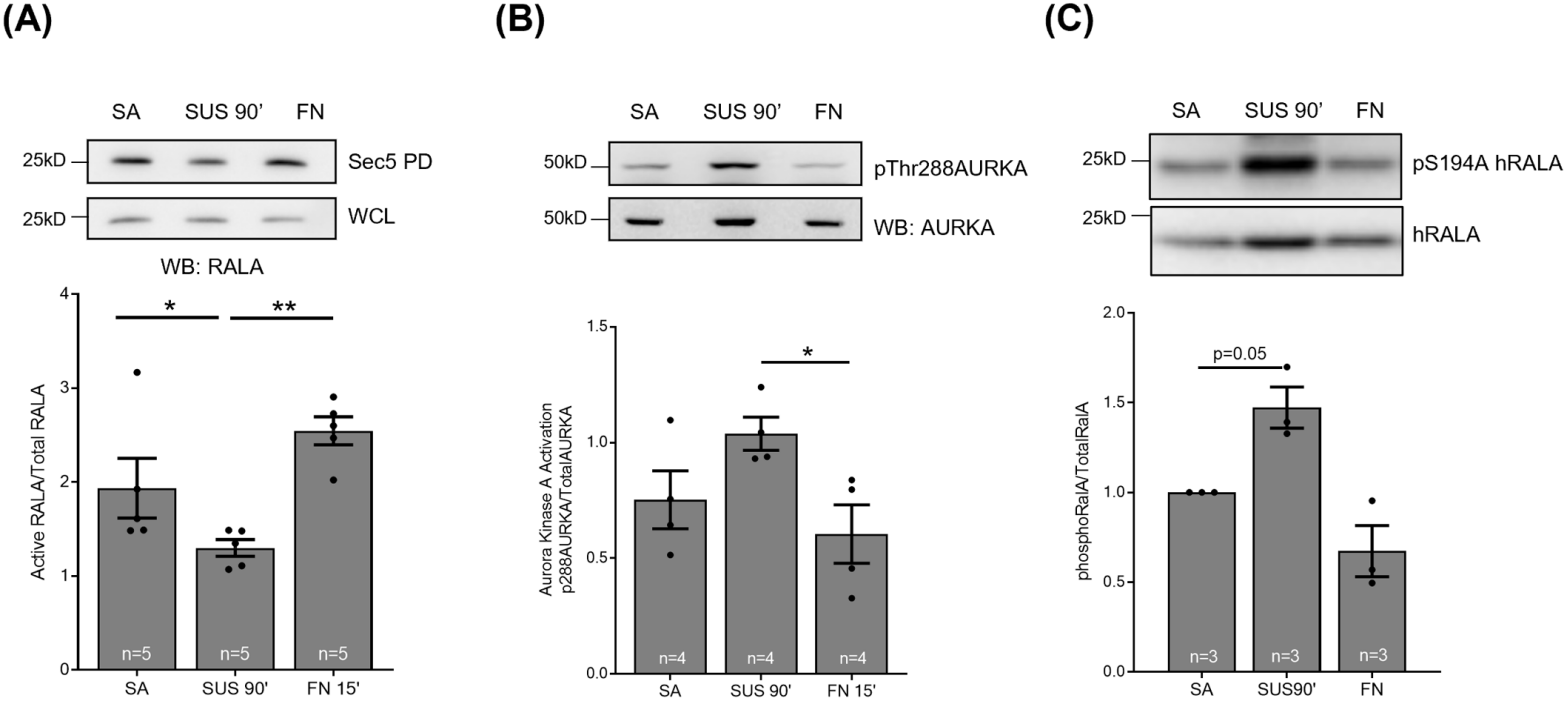
**Regulation of RALA and AURKA in early adhesion in WTMEFs.** Western blot detection (upper panel) and quantitation (lower panel) of (A) active RALA pulled down by GST-Sec5 (Sec 5), and the total RALA in whole cell lysate (WCL) from serum-starved WT-MEFs that are stable adherent (SA), suspended for 90 min (SUS 90′) and re-adherent on fibronectin for 15 min (FN 15′) probed for RALA (WB : RALA). The graph represents the percentage of active RALA calculated as described in methods from four independent experiments. (B) These lysates were probed to detect the phosphorylation of Threonine 288 residues of AURKA (pThr288AURKA) and total AURKA. (C) Cell lysates made from human RALA expressing serum starved WT-MEFs stable adherent (SA), suspended for 90 min (SUS 90′) and re-adherent on fibronectin for 15 min (FN 15′) were probed for phosphorylation on Serine 194 residues of human RALA (pSer194hRALA) and total expressed human RALA from four independent experiments. The graph represents the mean±s.e. Statistical analysis of data normalized to CON was done using the paired *t*-test, otherwise was done using Mann–Whitney test, and *P*-values, if significant, are represented in the graph (*P<0.05).

Earlier studies from the lab showed RALA activation to be regulated by cell-matrix adhesion (Balasubramanian et al., 2010; Pawar et al., 2016). Serum-deprived mouse fibroblasts, when held in suspension for 90 min, showed a distinct drop in RALA activation [when pulled down using the GST-Sec5 Ral binding domain (RBD)] that recovers on re-adhesion (Fig. 3A). Interestingly, AURKA activation (pThr288 AURKA) in these cells increases on loss of adhesion and is restored on re-adhesion (Fig. 3B). This suggests that AURKA and RALA activation could be inversely correlated in these cells. We hence asked if this AURKA regulation reflects on RALA S194 phosphorylation in these cells. The anti-phospho-RALA Antibody (Serine 194 residue) (Merck-Millipore, 07-2119), being specific to human RALA, does not allow for detecting mouse RALA phosphorylation. We, therefore, expressed human RALA in WTMEFs, which showed a distinct increase in S194 RALA phosphorylation on the loss of adhesion, which is restored on re-adhesion (Fig. 3C). This suggests adhesion dependent regulation of AURKA activation can indeed regulate RALA phosphorylation (pS194 RALA). This negatively regulates RALA activation in response to loss of adhesion (Fig. 3). The relationship between RALA phosphorylation and activation in earlier studies has been looked at using AURKA mutants in MDCK cells (Wu et al., 2005) or RALA phospho-mutants in HEK-TtH cells (Lim et al., 2010). These show RALA phosphorylation to be a positive regulator of RALA activation (Lim et al., 2010; Wu et al., 2005). Together, they suggest that AURKA-RALA crosstalk and its effect on RALA activation could be cell-type and context-dependent. The anti-phospho-RALA Antibody (Serine 194 residue) (Merck-Millipore, 07-2119) is seen to lack specificity in detecting pS194 RALA in some cancer cells, which could further complicate the interpretations made. Hence, the most effective way to overcome these limitations and test the role of RALA S194 phosphorylation in RALA function would be to evaluate RALA phosphomutant (S194A and S194D) expressing cells.

### Role of RALA and its phosphorylation in RALA activation

Verified RALA KO clones for MCF7 and MiaPaCa2 (clone 2) were used to make stable reconstitutions with WT-RALA, S194A-RALA (phosphodeficient) and S194D-RALA (phosphomimetic). While the S194A-RALA (phosphodeficient) mutant is effective in blocking phosphorylation in previous studies (Neel et al., 2014; Wu et al., 2005), the S194D phosphomimic mutant in one reported study was seen to behave like the S194A mutant (Lim et al., 2010). These RALA mutants were made by SDM PCR and were confirmed by sequencing (Fig. S3A) and PCR amplification using reverse primers ending at the mutation site (Fig. S3B).

**Fig. 4.**
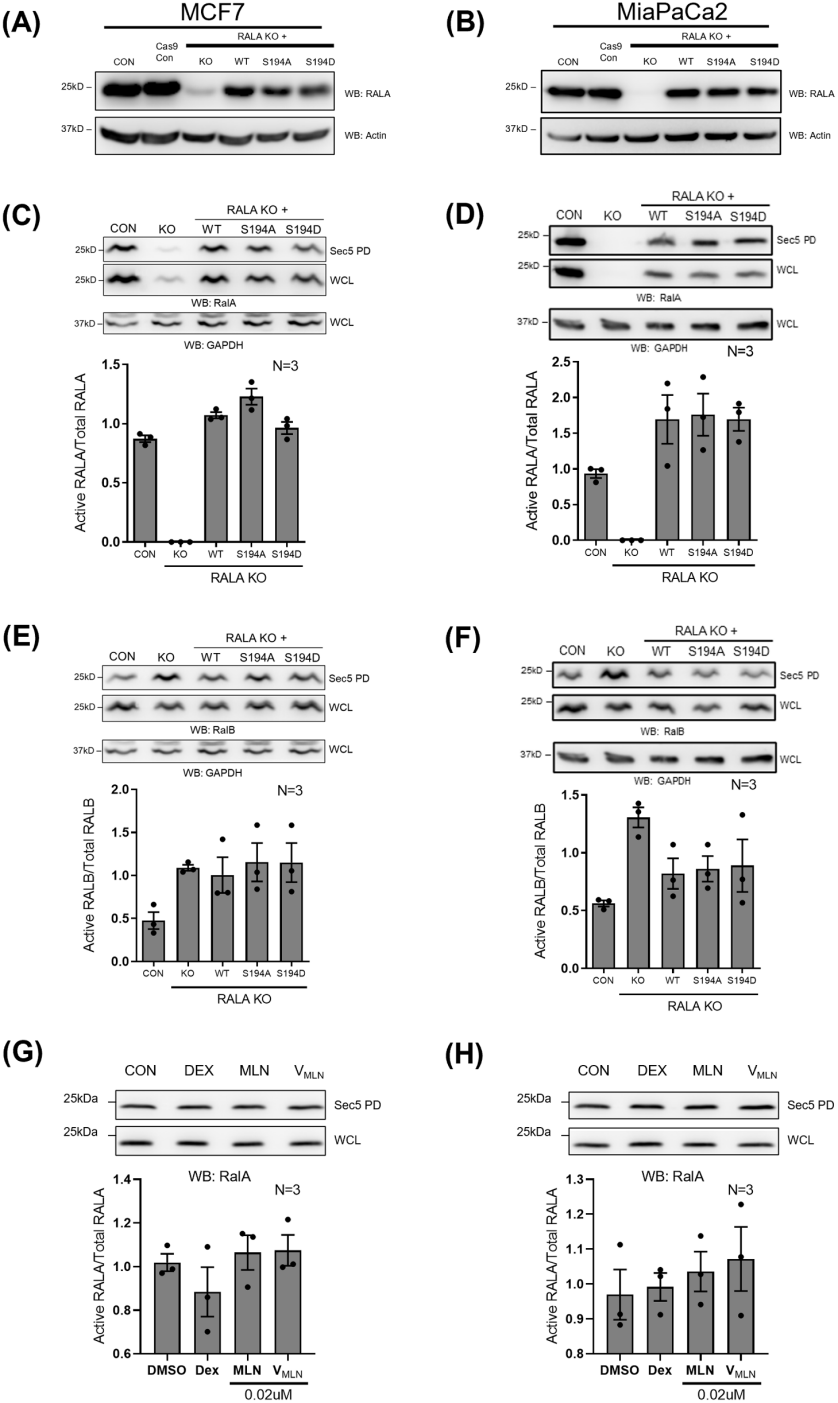
**Role of RALA and its S194 phosphorylation in regulating RAL activation in MCF7 and MiaPaCa2 cells.** (A,B) Western blot detection of RALA (WB: RALA) and actin (WB: actin) in cell lysates of MCF7 and MiaPaCa2 control (CON), RALA KO (KO) and RALA KO cells reconstituted with RALA mutants (WT/S194A/S194D). MCF7 Lysates were further probed for additional proteins of interest, with the results shown in Fig S3C. Blots were reused where required, and the RALA and actin blots from A are also shown for comparison in Fig. S3C. (C,D,E,F) Western blot detection (upper panel) and quantitation (lower panel) of active RALA and active RALB (in samples incubated with GST-Sec5, which pulls down active RALA and RALB from the same lysate), as well as total RALA and total RALB from lysates of control (CON), Cas9 control (Cas9), RALA KO (KO), WT-RALA (WT), S194A-RALA (S194A) and S194D-RALA (S194D) mutant reconstituted MCF7 (C,E) and MiaPaCa2 (D,F) cells. The same GAPDH blot is shown in C and E as the common loading control for RALA and RALB in the MCF lysates, and the same GAPDH blot is shown in D and F as the common loading control for RALA and RALB in the MiaPaCa2 lysates. (G,H) Western blot detection (upper panel) and quantitation (lower panel) of active RALA and total RALA from lysates of MCF7 and MiaPaCa2 cells treated with DMSO (CON), empty nano-vesicle scaffold (DEX), 0.02μM Free MLN and 0.02μM V_MLN_. The graphs represent the ratio of active to total protein (RALA and RALB) as mean±s.e. from at least three independent experiments. Statistical analysis was done using the Mann–Whitney test, and *P*-values, if significant, are represented in the graph (*P<0.05, **P<0.01, ***P<0.001).

The retroviral-mediated stable expression of WT-RALA and RALA mutants (S194A/S194D) in RALA KO MCF7 and MiaPaCa2 cell lines were comparable when detected with total RALA antibody (Fig. 4A and B). Their expression levels were less than endogenous RALA in untreated control cells (CON) (Fig. 4A and B). Detection of pS194RALA band in MCF7 mutants using phospho-RALA antibody (where detection is specific) also confirmed the mutation at S194 residue (Fig. S3C). GST-Sec5 pulldown of active RALA from reconstituted MCF7 and MiaPaCa2 cells showed no significant difference in the activation status of WT-RALA, S194A-RALA and S194D-RALA mutants (Fig. 4C and D). This suggests that RALA S194 phosphorylation does not affect RALA activation in RAS-independent (MCF7) or RAS-dependent (MiaPaCa2) cancer cell lines. RALB activity detected similarly was also unaffected by RALA mutant reconstitution of MCF7 or MiaPaCa2 cells (Fig. 4E and F). We did see a modest, non-significant increase in RALB activation upon RALA KO in both MCF7 and MiaPaCa2 (Fig. 4E and F).

As an additional method of confirming if RALA phosphorylation does not affect RALA activation in these cancers, we asked if V_MLN_-mediated inhibition of AURKA (that was seen to differentially affect RALA phosphorylation in MCF7 versus MiaPaCa2 cells) (Fig. 2C) affects RALA activation differently (Fig. S3D). When compared to respective controls (untreated CON, DEX control), V_MLN_ and free MLN8237 (MLN) treatment did not affect RALA activation in MCF7 or MiaPaCa2 cells (Fig. 4G and H). This agrees with the RALA mutant data in both cell types. Whether RALA phosphorylation could affect RALA function independent of its activation remains open.

### Role of RALA and its phosphorylation in tumor formation

**Fig. 5.**
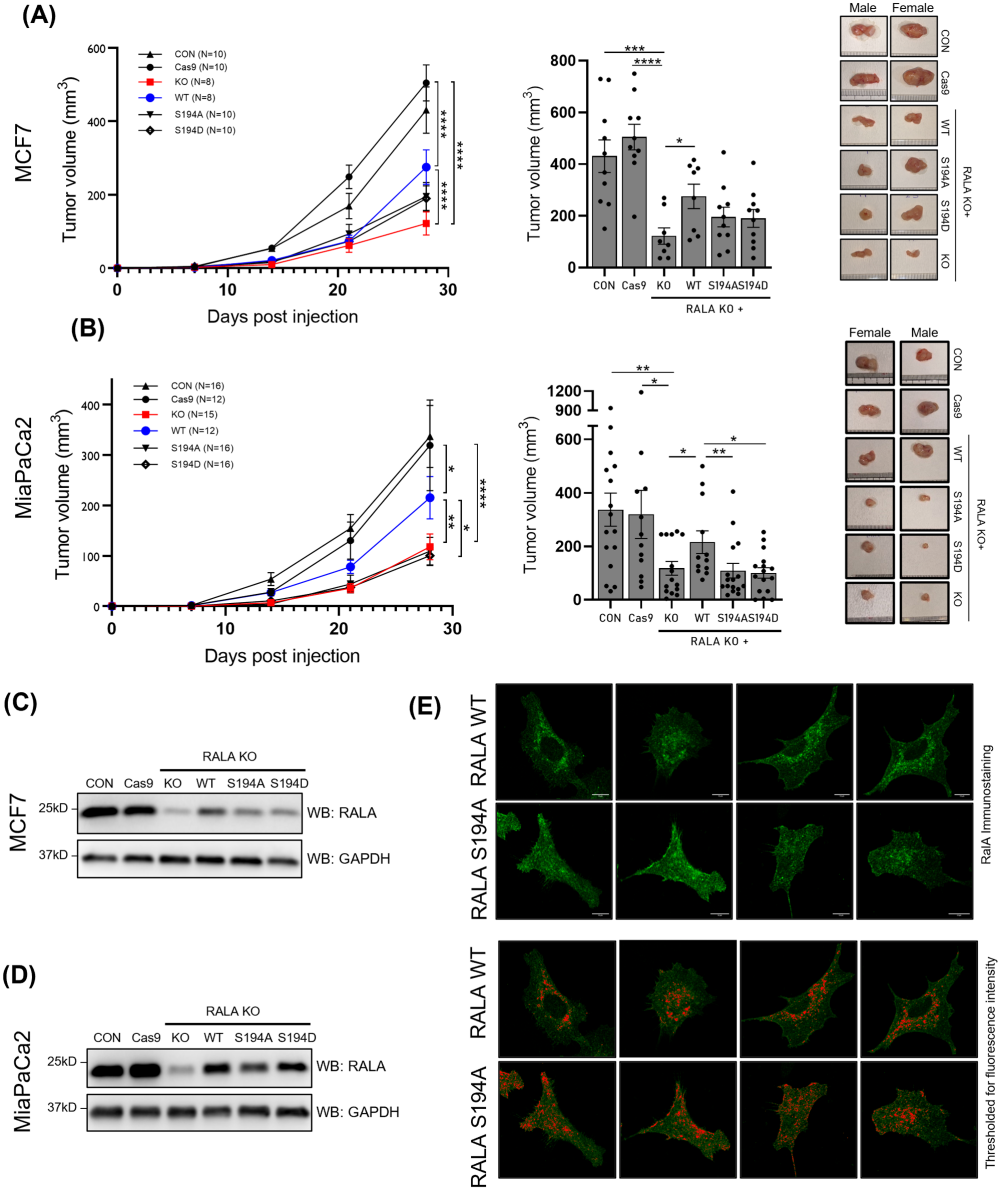
**Role of RALA and its S194 phosphorylation in regulating tumor formation and RALA localization in MCF7 and MiaPaCa2 cells.** Graph showing mean tumor volume (mm^3^) ±s.e. over 28 days and at 28 days (bar graph) for tumors made by MCF7 (A) and MiaPaCa2 (B) control cells (CON) (▴), Cas9 treated cells (Cas9) (•), RALA KO cells (KO) (▪) or RALA KO cells expressing WT-RALA (WT) (•) or S194A-RALA (S194A) (▾) or S194D-RALA (S194D) (♢) mutant cells. Statistical analysis was done using two-way ANOVA comparing mean of all groups. Statistical analysis was done for bar graphs using Student's *t*-test (for MCF7) and Mann–Whitney test (for MiaPaCa2) depending on distribution of data. *P*-values if significant are represented in the graph (*P<0.05, ****P<0.0001). (C,D) Western blot detection of RALA and GAPDH from harvested tumor lysates (protein equivalent) of control cells (CON), Cas9 control (Cas9), RALA KO (KO) and RALA mutants (WT, S194A and S194D) in MCF7 (C) and MiaPaCa2 (D) cells. At the same time, MiaPaCa2 lysates from the experiment in D were also probed for AURKA (presented in Fig. S3E along with the GAPDH blot) to evaluate the effect of RALA S194 mutation on AURKA expression in tumors. (E) Immunofluorescence detection of RALA in MCF7 RALA KO cells reconstituted with WT-RALA (top panel) and S194-RALA mutant (bottom panel). Fluorescence images were comparably thresholded, and intensities above the set threshold were marked in red to highlight their localization.

The best evaluation of RALA function in cancers stems from its ability to support tumor formation. Both MCF7 and MiaPaCa2 cells on RALA KO form significantly smaller tumors than control cells (Fig. 5A,B). This suggests the tumorigenesis of these cancer cells to be RALA-dependent. Evaluating the tumor-forming ability of WT-RALA expressing MCF7 and MiaPaCa2 cells showed a significant increase in tumor size, confirming the role of RALA in MCF7 and MiaPaCa2 tumorigenesis (Fig. 5A,B). This rescue being partial could be a reflection of the WT-RALA levels in reconstituted KO cells (MCF7 and MiaPaCa2) being lesser than endogenous RALA levels (in CON and Cas9-CON cells) (Fig. 5A,B,C,D). Also, we did not see any visible difference in the expression levels of AURKA in tumors formed by RALA KO and S194 mutant cells of both MCF7 and MiaPaCa2 (Fig. S3E). Together, this highlights the importance of RALA in tumor formation for both RAS-dependent MCF7 and RAS-independent MiaPaCa2 cells.

Tumors formed by MCF7 and MiaPaCa2 S194A-RALA (phosphodeficient) mutant reconstituted cells were comparable to their RALA KO tumors in size (Fig. 5A,B). They show that RALA S194 phosphorylation is indeed required for RALA-dependent tumor formation. In RAS-dependent MiaPaCa2 cells, the S194A-RALA (phosphodeficient) mutant cells were similar to the RALA KO cells in the size of the tumors they made (Fig. 5B). This suggests the RALA S194 phosphorylation is required for tumor formation in these cells. In RAS-independent MCF7 cells, the S194A-RALA (phosphodeficient) mutant cells behaved slightly differently. Their tumor sizes were marginally (not significantly) bigger than the RALA KO tumors and marginally (not significantly) smaller than the wild-type (WT)-RALA reconstituted tumors (Fig. 5A). This suggests the tumor formation in MCF7 could be marginally less dependent on RALA S194 phosphorylation. It hence supports earlier observations (Lim et al., 2010) that indicate the involvement of RALA phosphorylation could vary across cancer cell types.

In both MCF7 and MiaPaCa2 cells, tumors formed by S194D-RALA (phosphomimetic) mutant reconstituted cells were comparable in their size to the S194A-RALA mutants (Fig. 5A,B). This could mean the S194D-RALA (phosphomimetic) mutant may not be as effective in mimicking RALA phosphorylation in both cell types.

The localization of RALA and its regulation by RALA S194 phosphorylation could, in the absence of any effect on RALA activation, be responsible for regulating tumor growth. In MCF7 cells, the WT-RALA showed distinct perinuclear localization and occasional localization in membrane ruffles. S194A-RALA mutant lacked this spatial clarity in cells, being more homogeneously distributed. Fluorescence images, when comparably thresholded and intensities above the set threshold were marked clearly showed this spatial difference in the localization of WT versus S194A-RALA mutant (Fig. 5E). This localization difference in MiaPaCa2 cells was difficult to evaluate as cells spread distinctly less. Together, these studies implicate S194 phosphorylation as vital for the role RALA has in tumor formation in MCF7 and MiaPaCa2 cells, independent of its activation and possibly regulated by its localization.

## DISCUSSION

Evaluation of the role of RAL in cancer has been understandably focused on malignancies with RAS mutation, such as lung and pancreatic cancers (Richardson et al., 2022). Inconsistency in RAL function across cancer types illustrates a need to thoroughly evaluate their contribution and regulation in cancers (Richardson et al., 2022). CRISPR-Cas9 mediated knockout (KO) poses some advantages to siRNA- and shRNA-mediated protein expression targeting, including less off-target effects (Smith et al., 2017). Despite some of these advantages, only one previous study in MDA MB231 cells uses CRISPR-Cas9 to target RALA and RALB (Thies et al., 2021) and evaluated the role of RALA but not RALB in promoting AIG and tumorigenesis in MDA MB 231 cells. The making and validation of CRISPR-Cas9 RALA KO of MCF7, T24, UMUC3 and MiaPaCa2 cells is a vital step in this direction. No significant change in RALB, AURKA, AURKB, AKT, ERK, and AMPK levels or their activation status makes these cells ideal for reconstitution and other studies. With issues in genotyping (as seen for SKOV3) and non-specific targeting of genes, validation of CRISPR-Cas9 knockouts and mutants becomes particularly important.

In evaluating the RALA S194 phosphorylation using anti-phospho-RALA antibody (Serine 194 residue) (Merck-Millipore, 07-2119) in multiple cancer cell lines, we discovered that pS194RALA detection in certain RAS-dependent cancer cell lines (T24, UMUC3 and MiaPaCa2) could be non-specific. Validated CRISPR-Cas9 RALA KO cells, siRNA-mediated RALA KD and V_MLN_ mediated AURKA inhibition, all showed pS194RALA detection in T24, UMUC3 and MiaPaCa2 to be non-specific. Similar non-specificity in pS194RALA detection using this antibody was previously observed in pancreatic cancer cells upon AURKA inhibition using MLN8237 and siRNA-mediated RALA KD (PANC1 cells) (Neel et al., 2014). This antibody detected a change in RALA S194 phosphorylation in RAS-independent (MCF7 and SKOV3) cancer cells and non-transformed WTMEFs. It would however be premature to assume that this difference in detection of pS194RalA seen is dependent on the presence of oncogenic RAS. Studies expressing RAS^V12^ in the RAS-independent cells could help ascertain its role. This finding does affect how we and other studies interpret changes (or lack thereof) in pS194RALA levels in cells.

We further tested the role of RALA S194 phosphorylation in the regulation of RALA activation in cancer cells, which has been poorly studied until now. Surprisingly, RALA S194 phosphorylation does not promote RALA activation in WTMEFs, MCF7 and MiaPaCa2. In WTMEFs, a decrease in AURKA activation and RALA S194 phosphorylation promoted RALA activation in early cell-matrix adhesion. RALA S194 phosphorylation, which does not affect RALA activation, is reported in earlier studies. In HEK2393 cells, WT AURKA expression increases RALA S194 phosphorylation and activation (Lim et al., 2010), while overexpression of kinase-active AURKAThr288 further increases RALA S194 phosphorylation without having any effect on RALA activation (Lim et al., 2010). In HEK-TER cells, the RALA S194A mutant showed a drop in RALA activity (Sablina et al., 2007), while in NIH3T3 cells, this drop was seen but to a lesser extent (Lim et al., 2010). No such evaluation with RALA mutants has been done in cancer cells till now, and our data for the first time shows RALA S194 phosphorylation does not affect RALA activation in cancer cells.

While the role of RALA in tumor formation is well established in the literature, how RALA S194 phosphorylation affects tumorigenesis is less clear and more variable (Lim et al., 2010). RALA knockdown and reconstitution of pancreatic cancer cells showed Capan-1 and HPAC tumors to be pS194RALA dependent but not CFPac-1 tumors (Lim et al., 2010). Our study adds both MCF7 and MiaPaCa2 to the list of RALA phosphorylation dependent tumors. All of these cancer cells, except MCF7, express oncogenic RAS, suggesting RALA S194 phosphorylation-mediated regulation of tumorigenesis need not depend on oncogenic RAS. It is also worth noting that when compared to RALA KO, the S194A tumors are of comparable in size in some (Capan-1 and MiaPaCa2) and slightly bigger in others (HPAC and MCF7), suggesting there could be additional subtle variations in their role and regulation in cancers.

In our studies, the RALA S194D mutant does not effectively mimic phosphorylation. Its effect on MCF7 and MiaPaCa2 tumors and RALA activation is comparable to that of the S194A mutant. This agrees with an earlier study in non-transformed HEKTtH cells that shows the S194D-RALA mutant to behave like the S194A mutant in regulating RALA activation (Lim et al., 2010).

Since we do not see the effect of S194A mutation on RALA activation in MCF7 and MiaPaC2 cells but see a significant inhibition of tumor growth, we can speculate the role this phosphorylation could have in tumor formation may stem from its effect on RALA localization. A study in HEKTtH RALA knockdown cells reconstituted with WT-RALA versus S194-RALA mutant suggests a change in their localization to internal membranes, as seen in fractionation studies. This study also indicates that S194 phosphorylation can influence the interaction of RALA with downstream effector molecules such as RALBP1 (Lim et al., 2010), which could affect its role in tumorigenesis. An independent study observed that RALA and RALBP1 localize to the mitochondria, which depends on RALA S194 phosphorylation (Kashatus et al., 2011). We found that in MCF7 cells, S194-RALA mutant indeed showed distinctly different localization than WT-RALA. How this localization can regulate tumor growth without affecting RALA activation is an open question. One possibility is that this change in RALA localization may affect its interaction with downstream effectors (Lim et al., 2010) to differentially regulate tumors. Being able to recruit and trap RALA phosphomutants at a specific site in cells could help evaluate the role localization has in mediating its role in tumors.

Despite the complex nature of its regulation, the need for Serine-194 phosphorylation of RALA to support tumor formation is evident. This regulation and its implications for tumorigenesis is mostly cell-type-specific. Since S194 phosphorylation is vital for RALA-dependent tumor formation, targeting this site can have therapeutic advantages. Whether AURKA targeting could be an effective way to block RALA-dependent tumor growth is thus a question worth addressing.

## MATERIALS AND METHODS

### Cell culture and reagents

MCF7, SKOV3, T24, UMUC3, MiaPaCa2 and HEK293T cells were obtained from ATCC or ECACC. SKOV3, UMUC3 and T24 cells were cultured in high glucose DMEM medium with 5% fetal bovine serum (FBS) and MCF7 and MiaPaCa2 cells were cultured in high glucose DMEM medium with 5% FBS, penicillin, and streptomycin (Invitrogen).

Human plasma fibronectin (F2006), DMSO (D2438), DAPI (D9542), sodium orthovanadate (S6508), sparfloxacin (56968) and Thiazolyl Blue Tetrazolium Bromide (MTT, M2128) were purchased from Sigma, and Phalloidin-Alexa-488 (A12379), Phalloidin-Alexa-633 (A22284) was from Molecular Probes (Invitrogen). Fluoromount-G was used to mount cells for imaging and was obtained from Southern Biotech (0100-01). Glutathione sepharose beads used for the Ral activity assay were from GE Healthcare (17075601). Crystal Violet was used for staining colonies in the AIG assay from Amresco (0528). Alisertib (MLN8237) was purchased from Selleckchem (S1133). BQU57 (Ral inhibitor, SML-1268), and AZD1152 (AURKB inhibitor, SML0268) were purchased from Sigma. The BCA protein estimation kit (23227) was purchased from Thermo Fisher Scientific. Nonidet P40 Substitute (68387-90-6) and RNAase-A (9001-99-4) were purchased from USB corporation. Trizol (15596018) was purchased from Ambion. Immobilon western blot substrate (WBKLS0500) was purchased from Millipore.

Antibodies used for western blotting include anti-phospho-aurora A (Thr288)/aurora B (Thr232)/ aurora C (Thr198) (2914), anti-AURKB (3094), anti-phospho AKTSer473 (9271) (1:2000 dilution), anti-phospho-FAK-Tyr397 (3283), anti-phosphop44/ p42 ERK1/2 (Thr202/Tyr204) (4370) (1:2000 dilution), anti-p44/p42 ERK1/2 (4695) (1:2000 dilution), anti-FAK (3285), anti-AKT (4691) antibodies were purchased from Cell Signaling Technology and used at 1:1000 dilution unless mentioned otherwise. Anti-AURKA (610939) and anti-RALA (610221, clone 8) were purchased from BD Transduction Laboratories and used at 1:1000 dilution. Anti-Phospho-RALA (Ser194) (cat.# 07-2119) was purchased from Millipore, and Anti-RALB was from R&D Laboratories (AF3204), both used at 1:1000 dilution. Anti-beta actin (Ab3280) antibody was purchased from Abcam and used at 1:2000 dilution. Secondary antibodies conjugated with HRP were purchased from Jackson Immunoresearch and were used at a dilution of 1:10,000.

### Making of nanovesicle encapsulated MLN8237 (V_MLN_) with rhodamine B

Amphiphilic dextran (DEX-PDP) was synthesized as reported earlier (Pramod et al. 2012) and used to encapsulate MLN8237 in the polysaccharide (dextran) vesicles as reported earlier by (Inchanalkar et al., 2018). The drug loading content (DLC) and drug loading efficiencies (DLE) were determined by dissolving a known amount of a lyophilized drug-loaded sample in methanol and estimating its drug content by absorption spectroscopy. The molar extinction coefficient of MLN8237 was determined as 76500 l mol^−1^ cm^−1^ in methanol. The DLC and DLE were determined as 0.40% and 56%, respectively.

### Cellular uptake of V_MLN_ by confocal microscopy

MLN8237 and Rh-B were encapsulated together in dextran vesicles as described by (Inchanalkar et al., 2018) and their DLCs and DLEs were determined to be 0.42% and 58% for MLN8237 and 1.4% and 64% for Rh-B, respectively. Incubating cancer cells with V_MLN,_ their cellular uptake was evaluated in detail earlier (Inchanalkar et al., 2018).

### MLN8237 and V_MLN_ mediated AURKA inhibition

Cells (0.5×10^5^ cells) were seeded in six-well plates for 24 h and treated with 0.02 μM MLN8237 as a free drug or in the nanovesicle (V_MLN_) for 48 h. In all inhibition experiments, DMSO (CON) and empty dextran scaffold (DEX) were used as were solvent controls for free MLN8237 and encapsulated MLN8237 (V_MLN_), respectively. At the end of 48 h, cells were lysed in 150 μl of Laemmli buffer, resolved by SDS PAGE and western blotting was performed to check pAURKA, AURKA, pS194RALA and RALA.

### RAL activity assay

Control, RALA KO and RALA mutant reconstituted cells (2.5×10^5^) were seeded in a 10 cm dish for 72 h with or without nanovesicle-encapsulated inhibitor (V_MLN_). As reported earlier, active RAL was pulldown with the GST-Sec5 RBD (Inchanalkar et al., 2018). Percentage of active RALA and RALB, was calculated using:

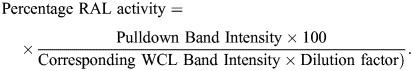
The dilution factor was calculated as the ratio of the amount of total cell lysate used for the pulldown (400 μl) and the amount of this lysate resolved by SDS PAGE as the WCL (24 μl WCL+6 μl 5× Lamelli buffer). The dilution factor was hence 400÷24=16.66. This was constant in all experiments.

### AIG assay

The effect of AURKA inhibition by a nanovesicle-encapsulated inhibitor (V_MLN_) on AIG was tested as described earlier (Inchanalkar et al., 2018). Cells were treated with V_MLN_ at 0.02 µM concentration for 10-14 days with fresh V_MLN_ added every third day.

### Making of CRISPR-Cas9 KO in cancer cell lines

We designed three sgRNAs against RALA, two were targeted against exon 2, flanking the translation start site (TSS) and one against exon 4. CHOP-CHOP and CRISPR KO online tools were used to design sgRNAs (Table 1) with high efficiency and low off-target binding. Low off-target binding was confirmed with the CCTOP online tool. sgRNAs were cloned into the pSpBBCas9-Puro vector using a restriction-based cloning method, as mentioned by Ran et al. 2013.

**Table 1.** sgRNA primer design

sgRNA primer	Target exon	Sequence
RALA sgRNA 2.1 FP	2	CACCGGCTTTACACAAAGTCATCATGG
RALA sgRNA 2.1 RP	2	AAACCCATGATGACTTTGTGTAAAGCC
RALA sgRNA 2.2 FP	2	CACCGATGGCTGCAAATAAGCCCAAGGG
RALA sgRNA 2.2 RP	2	AAACCCCTTGGGCTTATTTGCAGCCATC
RALA sgRNA 4.0 FP	4	CACCGCAGTGGAATGTTAACTACGTGG
RALA sgRNA 4.0 RP	4	AAACCCACGTAGTTAACATTCCACTGC

To make CRISPR-Cas9 KO, 1×10^5^ cells were seeded in 6 cm dish for 24 h. After 24 h, cells were transfected with 1 µg of empty pSpBBCas9-GFP vector and 1 µg of sgRNAs cloned in pSpBBCas9-Puro vector. After 48 h of transfection, we sorted the cells using GFP as single or double cells in 96-well plates using BD FACSAria. Cells were allowed to grow in 10% media till they formed colonies. Colonies were split and cultured in 48-well plates as duplicates. Colonies were screened using western blotting for RALA and Actin. Positive clones were expanded to 6 cm dishes and genotyping confirmed KO.

### Genotyping of RALA KO cells

For genotyping, cells from a confluent well of 48-well plate were lysed in 200 µl of tail lysis buffer and incubated with 2 µl of 20 mg/ml proteinase K and 2 µl of 10 mg/ml RNAse A overnight at 55°C (in a water bath). Proteinase K and RNAse A were denatured by heating at 95°C for 20 min. An equal amount of phenol: chloroform: isopropanol (PCI) was added to this vortex for 1 min and spun at 16,000 **g**/4°C/30 min. The supernatant was collected in a fresh tube, and an equal amount of isopropanol was added. The mix was kept at −20°C overnight, spun at 16,000 **g**/4°C/30 min and supernatant was discarded. The DNA pellet was washed with 100 µl of ethanol twice at 7500 **g**/4°C/30 min and the pellet was air dried. The DNA was then resuspended in Tris-EDTA buffer or NFW. 0.5 µg of DNA was used for genomic PCR in standard conditions. Primers were designed (Table 2) to bind at the exon-intron boundary and were used to amplify exon 2 and exon 4 (amplicon size: 200 bp), and PCR product was resolved on 2% agarose gel.

**Table 2.** Genotyping primers

sgRNA primer	Target exon	Sequence
hRALA exon2 gFP1	2	ACTGAGCATTACTTATTCTTTCATTC
hRALA exon2 gRP1	2	TAGCACTTACCTCATCGTACA
hRALA exon2 gFP2	2	TCCTTTGGTGAAAACTGAGACAC
hRALA exon2 gRP2	2	AAAATTAGCACTTACCTCATCGT
hRALA exon4 gFP1	4	TCTTATCCAGGGAGCAGATT
hRALA exon4 gRP1	4	GAGTCACGTGTTACCTTGTC
hRALA exon4 gFP2	4	TTTCATTTTCTCTTATCCAGGGAGC
hRALA exon4 gRP2	4	AACATTCCACTGCTCAGCTCT

### Reconstitution of RALA (WT/S194A/S194D) in RALA KO cells

RALA (WT/S194A/S194D) mutants were clones in pBABE puro vector and cloning was confirmed by PCR amplification using reverse primers ending at the mutation site (Table 3). For reconstitution, HEK293T cells were seeded 10×10^5^ cells in 10-cm dish. 24 h after cell seeding, cells were transfected with 2 µg of pGagPol, 3 µg of pVSVG, 7.5 µg of pBABE puro with RALA (WT/S194A/S194D) or empty pBABE GFP and 37.5 µl of lipofectamine 2000. 48 h after transfection, 2 ml of fresh media was added to cells. 60 h after transfection, media (virus titer) was collected and passed through 0.4 micron filter. 1×10^5^ RALA KO cells, were seeded in a six-well plate 36 h after transfection of HEK293T cells. 3 ml virus titer, 1 ml fresh media and 4 µl of polybrene (10 mg/ml) were added to the RALA KO cells 24 h after seeding. Cells were allowed to grow for 48-60 h (till we see transduction with the pBABE GFP vector). Transduced cells were selected with 1 µg/ml puromycin for 48-72 h until all cells in the control plate (transduced with empty pBABE GFP) died. After selection, the media was changed, and cells were allowed to recover for 24 h. After this, cells were transferred to 24-well plate and western blotting was performed to check the expression of RALA.

**Table 3.** RALA mutant screening primers

Screening primer	Sequence
RALA S194A screen RP	CTGATTCTCTTGGCTAAAAGC
RALA S194D screen RP	CTGATTCTCTTGGCTAAAATC

### Immunofluorescence assay

48 h after seeding on glass, cells were fixed with 3.5% paraformaldehyde for 15 min. Cells were permeabilized with PBS containing 5% BSA and 0.05% Triton-X-100 for 15 min and blocked with 5% BSA for 1 h at room temperature, followed by incubation with 1:500 mouse anti-RALA (BD Transduction laboratories) antibodies in 5% BSA at 4°C overnight. Cells were finally stained with 1:1000 diluted secondary antibodies (anti-mouse Alexa-488) or 1:500 diluted phalloidin-Alexa 594 for 1 h at room temperature. All incubations were done in a humidified chamber. Washes were done with 1× PBS at room temperature. Stained and washed coverslips were mounted with Fluoromount-G (Southern Biotech) and imaged using a Zeiss LSM780 multiphoton microscope with a 63× objective lens.

All image processing was done using FIJI ImageJ software. Initially, the brightness and contrast of all images were adjusted to have a comparable signal intensity. Following this, image thresholding was done to look at the localization RALA in cells.

### siRNA-mediated RALA knockdown in cancer cell lines

Seed 3×10^5^ cells per well in six-well plate for 24 h. After 24 h, transfected cells with 50 pmol of RALA siRNA (Table 4) and 3 µl of Lipofectamine RNAimax. After 24 h, change media and repeat transfection. 48 h after second transfection, cells were lysed for western blotting to confirm the Kd.

**Table 4.** siRNA for RALA knockdown

siRNA	Sequence
RALA	AAGGCAGGTTTCTGTAGAA
RALA-NB	AAGGCAGGTTTCTGTAGAA

### Mice xenograft

All animal procedures and numbers were approved by CPCSEA, New Delhi and Institutional Animal Ethics Committee (IAEC) (Approval number IISER_Pune/IAEC/2022_01/14). Animals used for experiments were provided by the NFGFHD, IISER, Pune. All experimental procedures were carried out in the presence of a veterinarian, and no estrogen pellets were used for this study.

For xenograft assay, an equal number of male and female NOD-SCID mice were used. One million cells were injected subcutaneously in 5–6-week-old mice. Mice were observed over 4-5 weeks, and mice weight and tumor size were measured weekly. At the end of the experiments, mice were euthanized exposing them to increasing concentration of CO_2_ in close chamber and tumors were harvested. 2 mm^3^ of the tumor was put in RIPA buffer, and cells were lysed using sonication. Debris was separated by centrifugation at 14,000 rpm, at 4°C for 15 min, and the supernatant was used for protein estimation using the BCA method. Laemmli lysates were prepared, and 20 µg of protein was used for western blotting.

### Statistical analysis

All analyses were done using Prism GraphPad analysis software. Statistical data analysis was done using the two-tailed unpaired Student's *t*-test, two-tailed paired *t*-test and, when normalized to respective controls, the two-tailed single-sample *t*-test. Mice tumor data were analyzed using two-way ANOVA (comparing columns within each row).

## Supplementary Material

10.1242/biolopen.061884_sup1 Supplementary information
