# Supplementary material for: CRISPR-Cas9 mediated RALA knockout and reconstitution: insights into the detection and role of RALA S194 phosphorylation in Ras-dependent and Ras-independent cancers
Source: Biol Open. 2025 Jul 21;14(7):bio061884. doi: 10.1242/bio.061884 (PMC12320973; doi:10.1242/bio.061884)
Supplement: Supplementary information [file biolopen-14-061884-s1.pdf]

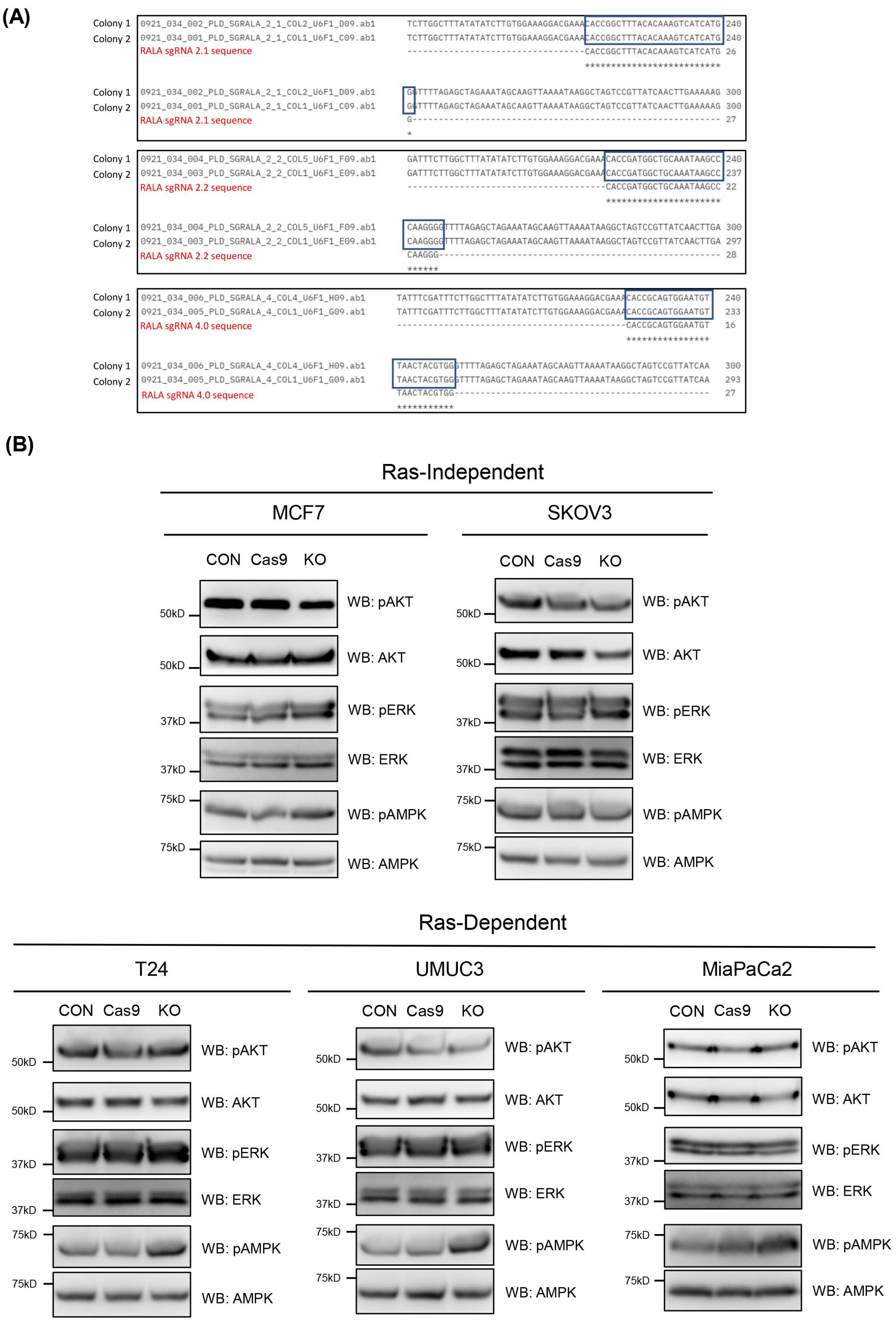
(A)
Colony 1
Colony 2
RALA sgRNA 2.1 sequence
RALA sgRNA 2.2 sequence
RALA sgRNA 4.0 sequence
(B)
Ras-Independent
MCF7
SKOV3
CON Cas9 KO
WB: pAKT
WB: AKT
WB: pERK
WB: ERK
WB: pAMPK
WB: AMPK
50kD
37kD
75kD
Ras-Dependent
T24
UMUC3
MiaPaCa2

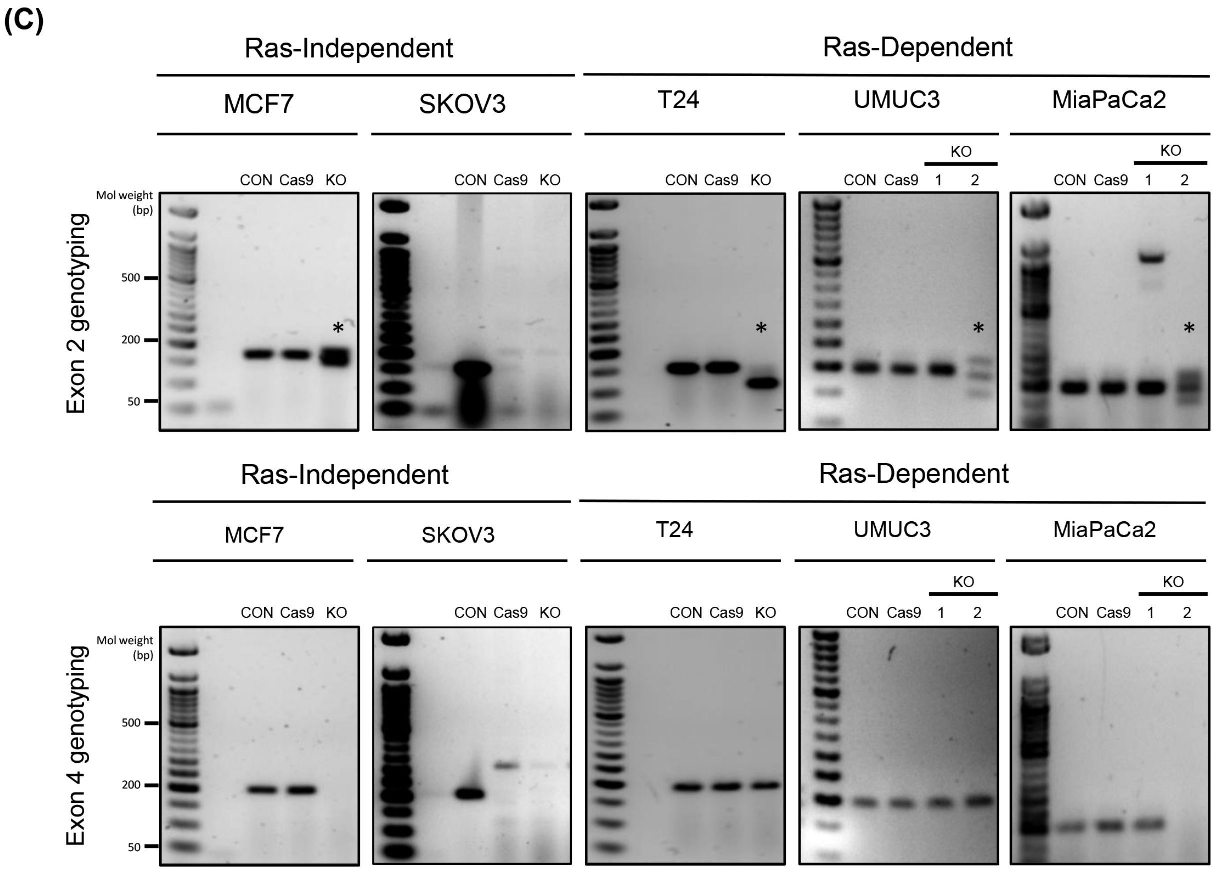

Fig. S1. (A) DNA sequence alignment of sgRNA clones in pSPCas9(BB)-2A-Puro vector from two colonies (colony1, colony2) each for RALA sgRNA (2.1, 2.2 and 4.0). The sequence for the sgRNA insert (shown in the box) was aligned with the sgRNA sequence used. Overlap is marked by asterix. **(B)** Western blot detection (WB) of AKT, ERK and AMPK in lysates of the untreated control (CON), Cas9 control (Cas9) and RALA knockout (KO) clones of RAS-independent (MCF7 and SKOV3) and RAS-dependent (T24, UMUC3 and MiaPaCa2) (single selected clone) cells. This was accompanied by the Western blot detection (WB) of active AKT (pAKT – pSer473 AKT), active ERK (pERK – pThr202/Tyr204 ERK1/2) and active AMPK (pAMPK – pThr172 AMPK). This detection evaluated the off-target effects of RALA KO on AKT, ERK and AMPK levels and activation. The lysates used were from the experiment shown in Fig. 1B and Fig. 2A. **(C)** Genotyping PCR amplicons from untreated control (CON), Cas9 control (Cas9), RALA knockout (KO) clone(s) of RAS-independent (MCF7 and SKOV3) and RAS-dependent (T24, UMUC3 and MiaPaCa2) cells. Exon 2 and 4 were amplified and resolved by agarose gel. Loss of amplicon or change in amplicon size (marked by asterisk) was detected in the gel.

Biology Open • Supplementary information

(A)

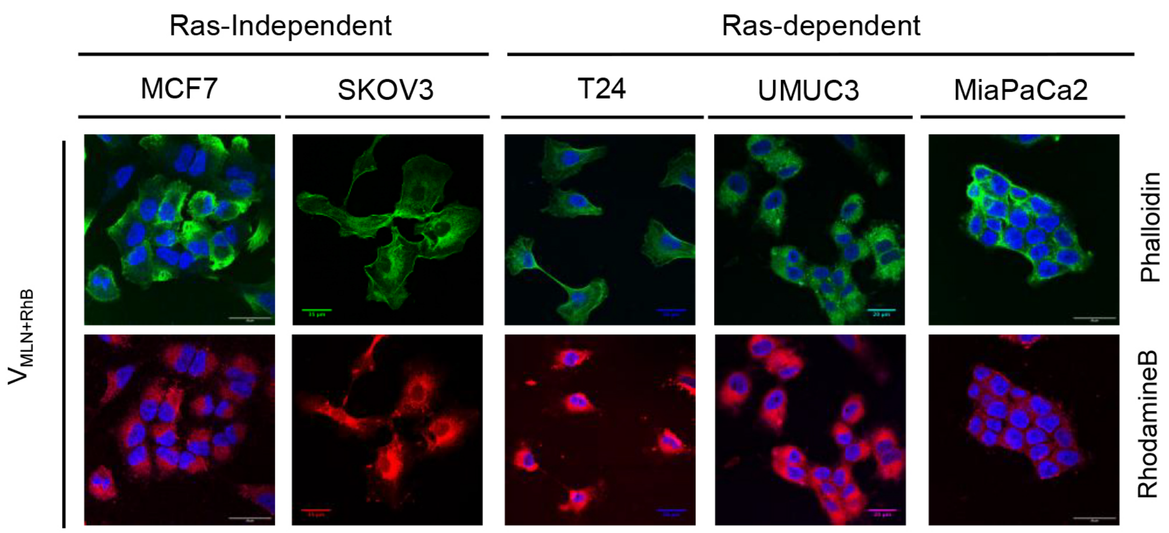

Fig. S2. (A) Uptake of Dextran nano-vesicle with MLN8237+ Rhodamine B ($V_{MLN+RhB}$) in RAS-independent **(MCF7 and SKOV3)** and RAS-dependent **(T24, UMUC3 and MiaPaCa2)** cancer cells were visualized by confocal microscopy in cells treated for 48 hours. The actin cytoskeletal network was stained with phalloidin conjugated to Alexa-488, and the nucleus was counterstained with DAPI.

(A)

RALA S194A cloning (AGT > GCT)

```
RALA WT       545 ACAGCAAAGAAAAGAATGGAAAAAAGAAGAGGAAAAGTTTAGCCAAGAGAATCAGAGAAA 604
S194A Col-2   553 ACAGCAAAGAAAAGAATGGAAAAAAGAAGAGGAAAGCTTTAGCCAAGAGAATCAGAGAAA 612
```

RALA S194D cloning (AGT > GAT)

```
RALA WT       542 AAGACAGCAAAGAAAAGAATGGAAAAAAGAAGAGGAAAAGTTTAGCCAAGAGAATCAGAG 601
S194D Col-2   551 AAGACAGCAAAGAAAAGAATGGAAAAAAGAAGAGGAAAGATTTAGCCAAGAGAATCAGAG 610
```

(B)

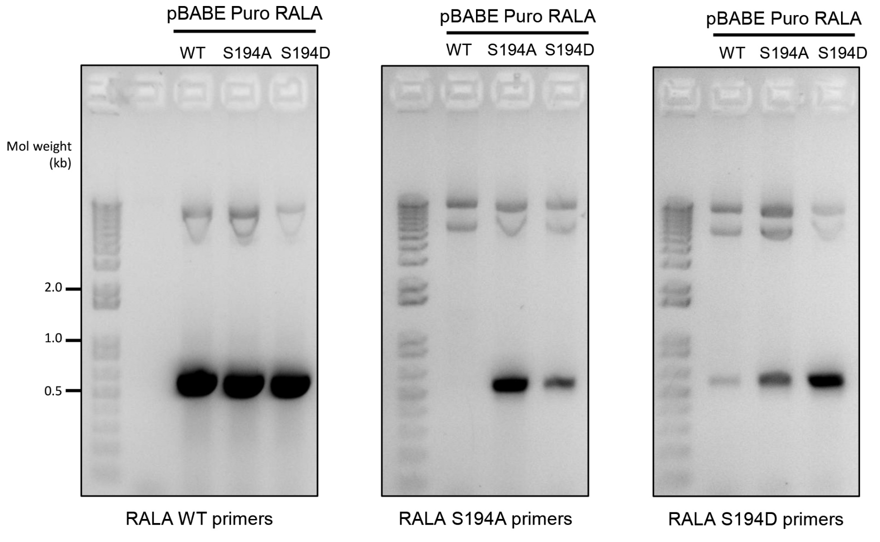

(C)

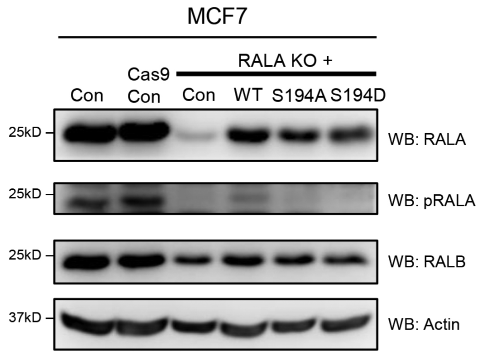

(D)

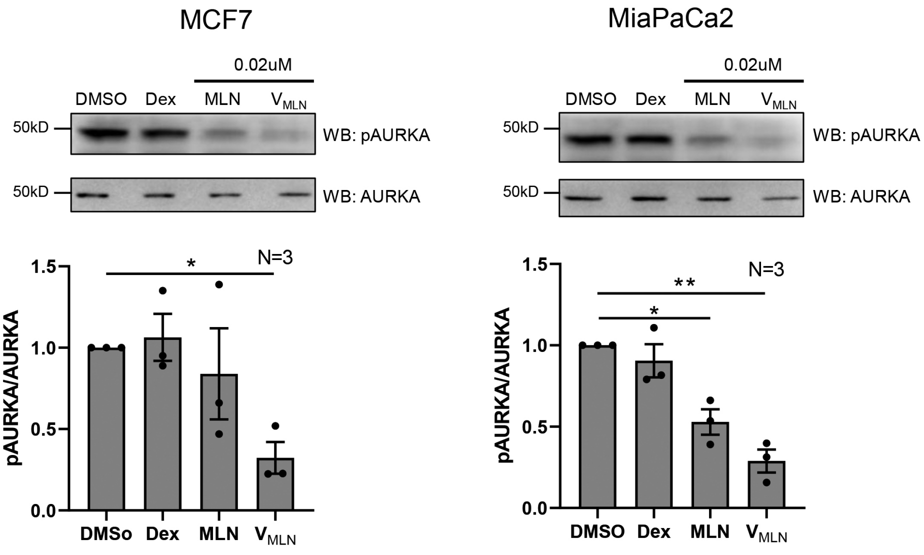

(E)

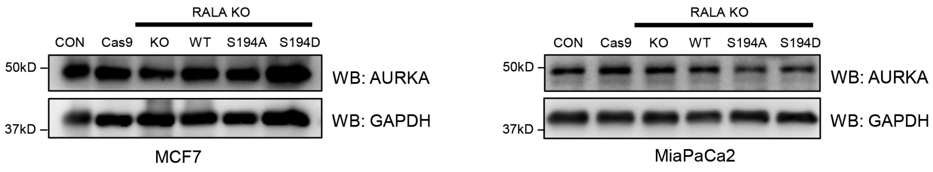

Fig. S3. (A) DNA sequences for S194A and S194D RALA mutants isolated from positive transformed colonies were compared to known WT RALA sequences (human mRNA NCBI database). The AGT base pair codon for Ser194 is marked in the WT RALA sequence and, when compared, shows distinct mutations in base pair codons for S194A (GCT) and S194D (GAT) mutants. **(B)** WT-RALA, S194A-RALA and S194D-RALA mutants cloned in the pBABE-Puro vector were amplified using RALA primers ending at the mutation site. Primers recognizing WT-RALA (AGT), S194A-RALA (GCT) and S194D-RALA (GAT) were used, and amplicons resolved on agarose gels. **(C)** Western blot detection of RALA S194 phosphorylation (pRALA), total RALA, RALB and Actin in MCF7 control (CON), Cas9 control (Cas9 CON), RALA KO (KO) and RALA KO expressing WT-RALA (WT), S194A-RALA (S194A)

Biology Open • Supplementary information

uantitation (lower and S194D-RALA (S194D) mutants. pRALA detection in these clones was part of the experiment presented in Fig. 4A and hence the RALA and actin blot from Fig 4A are shown here for comparison. This section evaluates the effect of S194 mutation on detection of pRALA in MCF7 cells. **(D)** Western blot detection (upper panel) and panel) of phosphorylation of Threonine 288 residue (pThr288 AURKA), total AURKA from whole cell lysates (WCL) of MCF7 and MIAPaCa2 cells treated for 48hours with DMSO (CON) and empty nano-vesicle scaffold (DEX), 0.02μM Free MLN (MLN) and 0.02μM encapsulated MLN (V_{MLN}). The graph represents the ratio of pAURKA/AURKA (normalized to CON as 1) as mean ± SE from three independent experiments. Statistical analysis was done using one sample T-test, and p values, if significant, are represented in the graph (* $p < 0.05$, ** $p<0.01$, *** $p<0.001$). **(E)** Western blot detection of AURKA and GAPDH from harvested tumor lysates (protein equivalent) of control cells (CON), Cas9 control (Cas9), RALA KO (KO) and RALA mutants (WT, S194A and S194D) in MCF7 and MiaPaCa2 cells. In MiaPaCa2 lysates, AURKA detection was part of the experiment presented in Fig. 5D and hence shows the GAPDH blot from Fig. 5D.

Biology Open • Supplementary information
